# Engraftment of Prevascularized, Tissue Engineered Constructs in a Novel Rabbit Segmental Bone Defect Model

**DOI:** 10.3390/ijms160612616

**Published:** 2015-06-04

**Authors:** Alexandre Kaempfen, Atanas Todorov, Sinan Güven, René D. Largo, Claude Jaquiéry, Arnaud Scherberich, Ivan Martin, Dirk J. Schaefer

**Affiliations:** 1 Clinic for Plastic, Reconstructive, Aesthetic and Hand Surgery, University Hospital Basel, 4031 Basel, Switzerland; E-Mails: rene.largo@usb.ch (R.D.L.); dirk.schaefer@usb.ch (D.J.S.); 2 Institute for Surgical Research and Hospital Management, University Hospital Basel, 4031 Basel, Switzerland; E-Mails: Atanas.Todorovjr@usb.ch (A.T.); guvens@stanford.edu (S.G.); arnaud.scherberich@usb.ch (A.S.); ivan.martin@usb.ch (I.M.); 3 Clinic for Oral and Cranio-Maxillofacial Surgery, University Hospital Basel, 4031 Basel, Switzerland; E-Mail: claude.jaquiery@usb.ch

**Keywords:** animal model, decellularized bone, tissue engineering, osteosynthesis vascularization, bone resorption

## Abstract

The gold standard treatment of large segmental bone defects is autologous bone transfer, which suffers from low availability and additional morbidity. Tissue engineered bone able to engraft orthotopically and a suitable animal model for pre-clinical testing are direly needed. This study aimed to evaluate engraftment of tissue-engineered bone with different prevascularization strategies in a novel segmental defect model in the rabbit humerus. Decellularized bone matrix (Tutobone) seeded with bone marrow mesenchymal stromal cells was used directly orthotopically or combined with a vessel and inserted immediately (1-step) or only after six weeks of subcutaneous “incubation” (2-step). After 12 weeks, histological and radiological assessment was performed. Variable callus formation was observed. No bone formation or remodeling of the graft through TRAP positive osteoclasts could be detected. Instead, a variable amount of necrotic tissue formed. Although necrotic area correlated significantly with amount of vessels and the 2-step strategy had significantly more vessels than the 1-step strategy, no significant reduction of necrotic area was found. In conclusion, the animal model developed here represents a highly challenging situation, for which a suitable engineered bone graft with better prevascularization, better resorbability and higher osteogenicity has yet to be developed.

## 1. Introduction

The gold standard for the treatment of large segmental bone defects is autologous bone transfer. However, due to the associated morbidity and low availability, an alternative with similar properties is direly needed. Bone substitute materials such as ceramics, polymers, bioglasses and many others have been developed [1,2]. Still, bone tissue is mechanically superior and contains many signals required for the coordinated resorption and reconstitution of host bone, called remodeling [3]. Therefore decellularized bone is in clinical and scientific use as a bone substitute [4,5,6]. Tissue engineering approaches introduce a cellular compartment that enhances the engraftment and assists bone tissue formation by generating a more physiological microenvironment. Specifically, the introduction of osteogenic cells such as osteoblast and osteoclasts enhances the formation and assists the remodeling of newly formed bone tissue [7]. Mesenchymal stromal cells from bone marrow and adipose tissue are recognized as a relevant source for osteoblast progenitors and have been widely used in tissue engineering to promote bone formation *in vitro* and *in vivo* [8,9,10]. Especially during bone surgery, the additional use of bone marrow derived mesenchymal stromal cells in an implanted graft is a natural consideration.

Clinical cases of large bone defects often entail a poor healing capacity, which makes an optimal performance of the osteogenic graft a necessity. In this context, vascularization is particularly recognized as a limiting factor for tissue engraftment and regeneration, and plays a central role for tissue engineered graft success or failure. An *in vitro* generation of vascular networks inside engineered osteogenic grafts has been previously described [11] but still cannot fully meet the critical needs related to the *in vivo* functionality of large grafts, especially in traumatic contexts. *In vivo* prevascularization strategies such as placing the grafts in the vicinity of clearly identified vessels or even creating a bundle of vessels around or through the grafts have been previously described [9,12]. Even within these subsequently pedicled grafts, the generation of vascular networks is a fragile process and much discussion is ongoing to identify what type of environment is most beneficial [13]. Some have suggested that an initial ectopic implantation and incubation of the engineered graft could be the best vascularization strategy prior to final orthotopic use [14,15]. To our knowledge, a relevant comparison between pedicled engineered grafts with different *in vivo* incubation procedures and a direct use of unpedicled grafts in a challenging orthotopic environment has not been demonstrated.

The goal of the present study is to evaluate three different prevascularization strategies of an engineered bone graft in a segmental bone defect model and to determine the most optimal outcome in terms of vascularization and bone formation. We prefabricated a tissue engineered osteogenic graft from a decellularized bone scaffold seeded with autologous bone marrow derived mesenchymal stromal cells (BMSC). To improve the clinical relevance, we utilized a novel segmental bone defect model in the rabbit humerus. This model is cost- and time-effective and mimics clinical challenges such as simulation of mechanical stress, the size of the defect and lack of internal splints in a single setting. This report focuses on the very early stages of graft survival through an investigation of the vascularization and immediate graft remodeling.

## 2. Results

**Figure 1:**
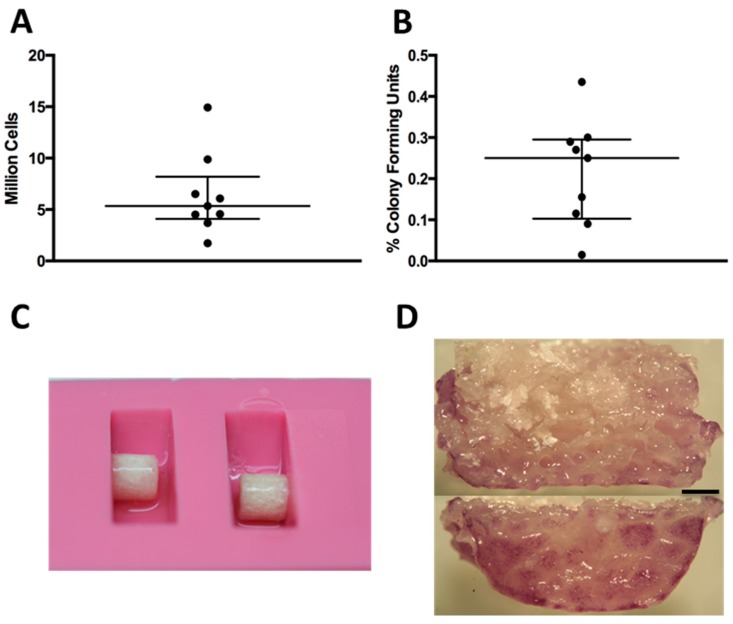
(**A**) Amount of nucleated cells extracted per bone marrow biopsy; each dot represents one donor; (**B**) Percentage of colony forming units per nucleated cells extracted from bone marrow biopsy; (**C**) Silicon mold with scaffold during seeding; (**D**) Tetrazolium (MTT) staining showing the distribution of cells along the periphery after seeding: the top image is a midline section, and the bottom is the outside surface. Black bar represents 1 mm.

Cell isolation yields and percentage of colony forming units from rabbit bone marrow aspirates showed donor variability (Figure 1A,B). The monolayer expansion of isolated primary BMSCs required 25.1 ± 5.4 days to reach approximately two million cells. The seeding efficiency of BMSCs onto the scaffolds using a fibrin hydrogel and a silicon mold (Figure 1C) was always greater than 99%. The cells were found distributed along the periphery of the scaffolds (Figure 1D).

**Figure 2:**
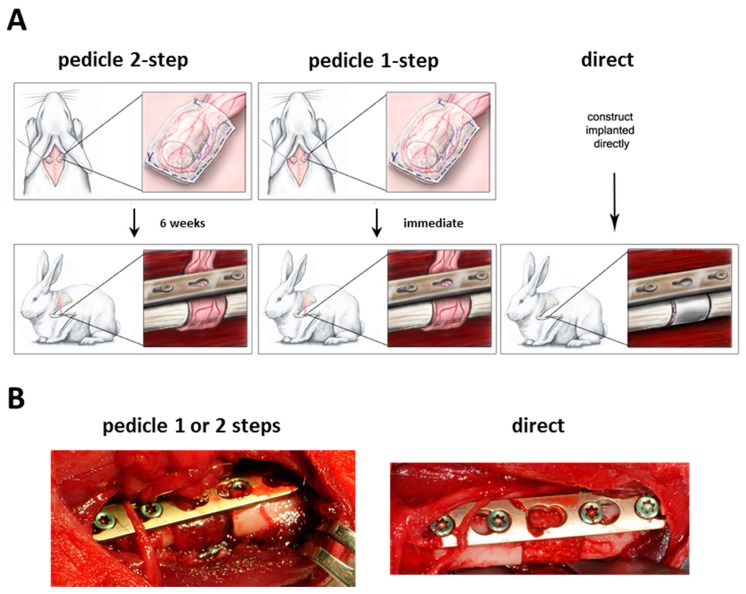
(**A**) Experimental set-up of the different groups; (**B**) Representative intraoperative images of the orthotopic defect.

The surgical operations were performed without significant complications according to the method described (Figure 2A,B). The pedicle 2-step group did not experience much scar formation and the recovery of the graft after six weeks was easily possible. Rotation of the graft in the pedicle 2-step and 1-step groups was feasible without any tension on the pedicle or defect site.

**Figure 3:**
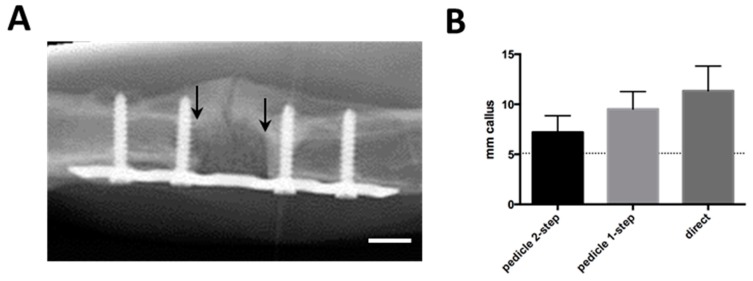

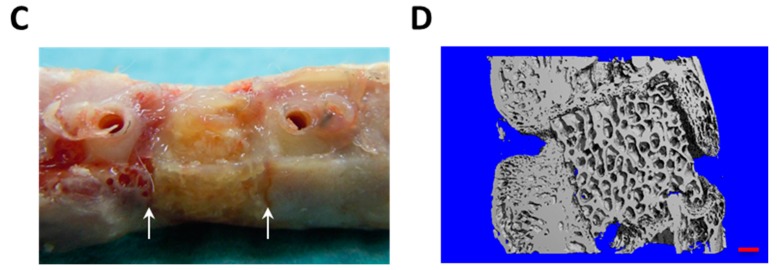
(**A**) Follow-up radiological image showing scaffold in orthotopical defect after 10 weeks. Callus formation is visible on the opposite side of the plate. Arrows indicate proximal and distal edges of defect, white bar represents 5 mm; (**B**) Radiological quantification of callus as seen in (**A**), represented as average mm of radioopaque mass at the proximal and distal edges of the defect. Dotted line represents normal bone diameter at the same location; (**C**) Macroscopical appearance after plate removal. Arrows indicate the edges of the defect; (**D**) Post-explantation microtomography. Red bar represents 1 mm. None of the groups displayed macroscopic or microradiographic evidence of fracture consolidation during the experimental period.

During the 12-week *in vivo* period, radiographs showed a variable amount of radiopaque mineralized callus tissue, which grew at both ends of the segmental defect (Figure 3A). There were no significant differences in callus formation between groups (Figure 3B). No bridging of the fracture or integration of the scaffold up to 12 weeks was observed in any of the experimental groups. The macroscopic assessment allowed the same observation (Figure 3C). A closer investigation using microtomography and 3D image reconstructions showed that the scaffold and surrounding bone and callus had very similar densities, yet there was no calcified connection between host and scaffold in any of the groups (Figure 3D).

**Figure 4:**
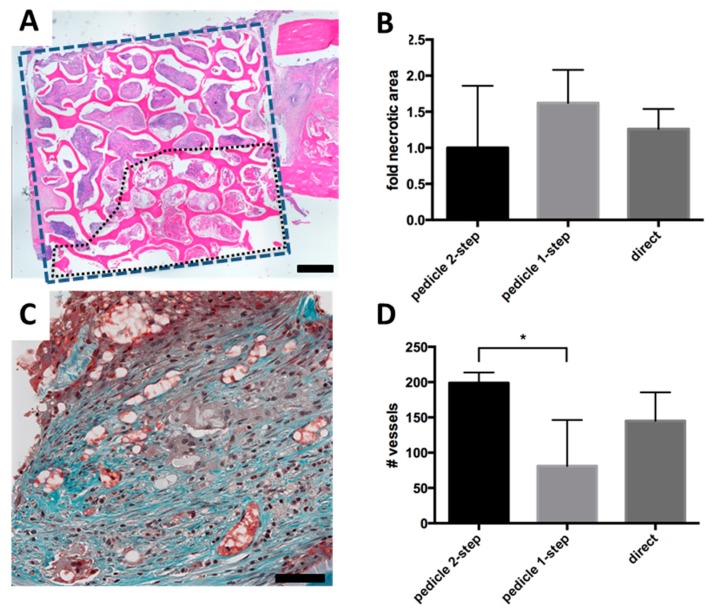

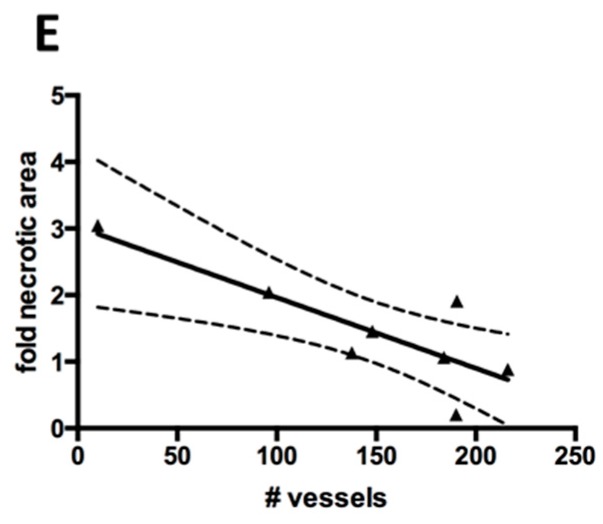
(**A**) Analysis of necrotic core formation based on hematoxylin and eosin (H&E) staining. Blue rectangle indicates the total area considered during quantification. Black dotted outline is an example of necrotic area. Black bar represents 1 mm; (**B**) Necrotic area observed in the experimental groups, represented as fold of the average necrotic area in the pedicle 2-step group; (**C**) Representative Goldner Trichrome staining with vessels appearing red in high magnification. Black bar represents 50 mm; (**D**) Average vessel number inside the scaffold (blue rectangle in **A**), counted by Goldner Tichrome staining. Significant difference is marked by *; (**E**) Correlation of vessel number and necrotic area for each sample. Black line represents linear regression fit and dotted lines represent 95% confidence intervals.

Histological analysis did not demonstrate new bone formation inside the scaffold. Instead there was a variable amount of cell and tissue necrosis (Figure 4A). Although not significant, there was a trend towards less necrosis in the pedicle 2-step group (Figure 4B). A quantification of the density of blood vessels showed that the 2-step group had significantly (*p =* 0.043) more vessel sections than the 1-step group (Figure 4C,D). The difference between the pedicle 2-step group and the direct group was not significant. We found a significant inverse correlation (*R*^2^ = 0.677, *p =* 0.012) between the necrotic area and the density of blood vessels (Figure 4E).

**Figure 5:**
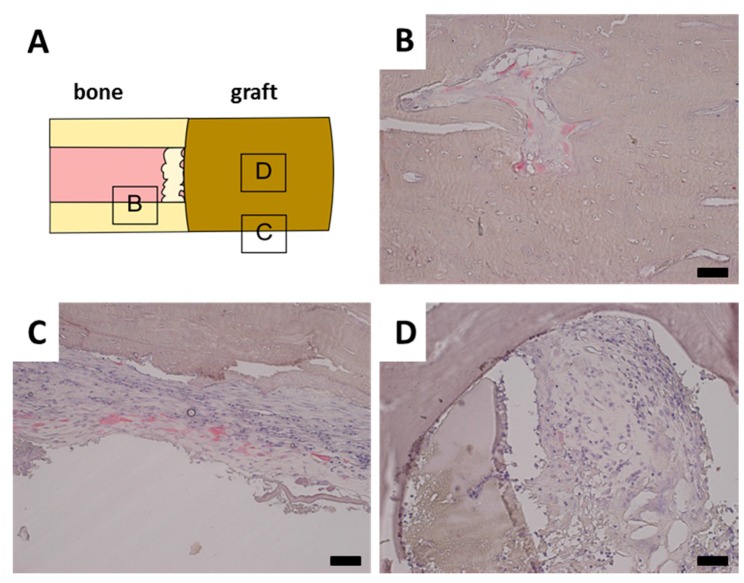
Representative tartrate resistant acid phosphatase (TRAP) stainings. Black bar represents 50 mm in each image. (**A**) Schematical display of TRAP staining location; (**B**) Normal bone has closely associated TRAP positive osteoclasts; (**C**) Periost around fracture displays increased presence of TRAP positive cells; (**D**) Tutobone scaffold with invading granulation tissue, however no closely associated TRAP positive cells.

A TRAP staining was performed to investigate the remodeling of the scaffold by osteoclasts. TRAP positive osteoclasts were found in normal trabecular bone adjacent to the defect site as well as in parts of the calcified callus (Figure 5B). TRAP positive cells were also observed inside the periost in the vicinity of the defect site (Figure 5C). However, no TRAP positive cells were observed in contact with the scaffold material in any of the samples, even though there was a clear presence of granulation tissue (Figure 5D).

## 3. Discussion

In the presented study, we have introduced a novel segmental defect model and tested a treatment based on a prevascularized, seeded graft. We could identify a promising prevascularization strategy in this setting, yet could not observe a significant reduction in necrotic area nor any bone formation inside the scaffold.

Our first approach was to implant the scaffold near a defined vessel and incubate it ectopically until a vascular network had developed. In a second operation, the pedicled vascular graft is then transferred into the defect. This favorable environment should facilitate the initial vascularization and thus enhance the performance of the graft, as suggested by Warnke *et al.* [14,15]. Our second approach was to insert both the BMSC-seeded construct and the surrounding vasculature directly into the orthotopic site. This approach would be clinically more attractive since it would require only one surgical intervention. However, the environment would be less favorable to vascularization due to increased inflammation and therefore the performance is expected to be more strongly dependent on the construct’s properties at implantation. The third approach was to just insert the construct into the bone defect and rely on the vascularization that should naturally occur from the neighboring orthotopic environment, including the surrounding musculature.

Only few animals per group (*n =* 3) were used in this study, in part because we expected a much greater difference between the treatment groups and a better outcome in terms of bone formation. However, according to the principles of 3R (reduce, refine, replace), it was deemed unethical to increase the animal number if the expected difference between the groups became too small.

We chose a simple way of identifying autologous rabbit BMSCs based on plastic adhesion and proliferation in culture medium. Since clean bone marrow aspirations were performed and a medium without specific factors favoring macrophages or other hematopoietic cells was used, only plastic adhesive, fibroblast-like BMSC could survive and proliferate [16]. Additionally, defined markers specific for rabbit BMSC are lacking and this precludes a more in depth analysis of the cell population. We decided against specifically labeling the BMSCs because our goal was to improve bone formation without alteration of the BMSCs. After monolayer expansion, we seeded 2 × 10^6^ BMSCs to have a consistent number across donors and a short *in vitro* expansion of the cells. We used a simplified method involving a silicon mold, which gave a relatively homogenous distribution of cells along the periphery of the scaffold. Although not pre-differentiated to osteoblasts, it was expected that BMSCs would be able to react to cues from the orthotopic environment or from the scaffold itself. Whether this process did not occur or whether it was inhibited by the local inflammation at the orthotopic site should be elucidated in future trials. Since histologically no bone formation occurred in any of the samples and instead a more or less pronounced necrosis was found, we deemed it non-informative to perform immunohistochemistry for bone proteins or PCR for osteogenic markers in the grafts.

A quantification of the vessel density inside the constructs revealed that an ectopic prevascularization resulted in a more than twofold higher blood vessel density, which was significant compared to the orthotopic prevascularization. However, the difference to the non-prevascularized graft was not significant. The vascularization inversely correlated with the necrotic area. The trend of vascularization and reduced necrotic area could indicate that for the present construct type roughly, 300 vessels per midline section would be required to optimally prevent necrotic core formation. The ectopic prevascularization group had an additional six weeks *in vivo*, which clearly could result in more vascularization and less necrosis than the other groups. However, even with improved vascularization, necrosis was not significantly different. Possibly, the overwhelming inflammation and mechanical stresses in an orthotopic environment would not only compromise new blood vessel formation but also lead to a failure of already existing vessels, resulting in severe tissue necrosis. Even a pre-conditioned vascular network would struggle to withstand such local stresses. Actual bone formation could require a longer time period after complete vascularization for the access of appropriate progenitors and thus osteogenesis in the graft.

Our chosen quantification method based on erythrocytes within tubular structures, offers the advantages that it is straightforward and does not require much additional processing of the samples. It does suffer from false-negative observations and a low sensitivity. Additionally, for a meaningful observation of the very center of the scaffold, only few central midline sections could be used. However, it was sufficient to show significant differences between experimental groups. Endothelial stainings such as CD31 or von Willebrand factor are prone to different types of error, such as the presence of vessel sprouts that are not connected to the vasculature or unspecific staining of bone marrow cells. For our purposes, vessels with proven perfusion were of greater relevance. Future improvements would be the perfusion with lectin or India ink, or most optimally the perfusion with radiological contrasting agents and subsequent analysis of the vascular tree using microtomography [17].

We did not observe a consolidation of the defect or a complete remodeling of the scaffold during the study period. This observation was in part expected due to the challenging nature of the bone defect. Callus formation at the proximal and distal edges was present in all groups, which indicated a limited stability of the osteosynthesis and variable animal activity. The callus preferably grew around the construct rather than through it, possibly a sign of a slow scaffold resorption. Although the quantification of bone through microtomography is considered a gold standard, its use in our study was excluded due to the complete lack of bone formation in the graft. Additionally this technique is very challenging when decellularized bone matrix is present, since it has in principle the same radiological density as bone. On the other hand, a quantification of callus formation was performed based on radiographs to ensure that groups did not differ significantly in stability of the osteosynthesis. Even though this method is less precise than microtomography, it allows a fast and easy non-invasive monitoring.

In this study, we chose the FDA-approved Tutobone^®^ scaffolding material because of its mechanical stability and routine applications in clinics as a bone substitute. Moreover, this scaffold consists of decellularized bone and therefore its calcium content and radiological density are very similar to native bone. It is expected to support bone formation in the engineered graft both through osteoconduction and osteoinduction. Only in the later stages would resorption eventually remove the graft and replace it with host-derived bone tissue [18]. Such a slow integration has already been observed in a clinical setting, where remnants of the scaffold have even been found after 11 months [19].

In the granulation tissue formed within the scaffold, no TRAP positive osteoclasts adjacent to the scaffold material were found. This was in contrast to the osteoclasts observed in the normal bone surrounding the defect. However, there was a considerable amount of TRAP positive cells in the periost closely associated to the defect site. In the callus tissue, TRAP positive osteoclasts were rare and only found in the vicinity of normal bone. Taken together, these results indicate that osteoclasts were only slowly migrating into the construct, but no resorption had started there yet. It is known that osteoclast precursors are found in the monocyte fraction of peripheral blood [20]. Therefore, a better vascularization might lead to a better access of these precursors to the construct, which likely does not release factors able to attract vascularization and monocytes by itself.

Rabbits are widely used in bone tissue engineering and are one of the most cost-effective pre-clinical animal models [21]. The size of the orthotopic defect allows a translation to small human bones, e.g., human metacarpals in hand surgery. Additionally, rabbits are immunocompetent and require the use of autologous cells and non-immunogenic material, a crucial requirement for clinical translation. Compared to larger animal models [22,23,24] or small-scale clinical trials [25,26], a good rabbit model can be a more valuable research tool if it can replicate all the necessary challenges. We chose the rabbit humerus as orthotopic implantation site because there is no possible internal splinting phenomenon (radius and ulna, tibia and fibula), no interosseous membrane to provide a source of osteogenic cells and no external fixation or casting required for stability such as in the femur. It was estimated by veterinaries and surgeons that 7 mm, representing 10% of the bone length, would be sufficiently challenging and would not spontaneously heal. In this setup, the plate stability may decrease during the experimental period and the implanted constructs may experience a significant fraction of the physiological mechanical stresses. These challenges are compounded by the size of the missing segment and the limited vascular supply on site. Although similar models have been proposed in literature [27,28,29], they have not combined the segmental humeral defect with plate stabilization and a significant vascular ingrowth distance to the core of the graft. We propose that this model is valuable for testing therapeutic strategies in the treatment of large, unstable bone defects.

In conclusion, this study presents a highly challenging animal model for the development of therapies for large segmental bone defects. We tested different prevascularization strategies and found indications that even a somewhat improved prevascularization could not prevent tissue necrosis in an orthotopic environment. An even more intensive prevascularization with the use of factors such as VEGF could be used in future. Additionally, a resorbable and osteoinductive scaffold, e.g., a collagen sponge with BMP2, could be used to improve bone formation. Lastly, better osteoblastic differentiation of BMSC and addition of osteoclasts could create a true osteogenic graft.

## 4. Experimental Section

### 4.1. Animals

All animal procedures were approved by the Swiss Federal Veterinary Office (BS2080; 1 January 2011, Basel, Switzerland) and conducted in accordance with the guidelines for care and maintenance of animals at the University Hospital Basel. A total of nine skeletally mature New Zealand White Rabbits (NZW, Charles River Laboratories, Kisslegg, Germany) with an average body weight of 2.29 ± 0.23 kg were used. For operative procedures, animals were anaesthetized by administration of 25 mg/kg ketamine (Ketaminol 5%, Veterinaria AG, Zürich, Switzerland) and 2.5 mg/kg xylazine (Narcoxyl 2% ad usus veterinarium, Veterinaria AG). Additional inhalation anesthesia during procedures was performed with isoflurane (Forene, Abbott AG, Baar, Switzerland). Analgesia was given by injection of 0.05 mg/kg buprenorphine (Temgesic, Essex Chemie AG, Luzern, Switzerland) and administered 2–3 times daily for the first 2 weeks. After this period, analgesia was administered when animals displayed signs of pain or distress. For all procedures, body temperature, heart rate, respiratory frequency and peripheral oxygenation (Pulse oximeter NPB-290, Nellcor Puritan, Bennett, Pleasanton, CA, USA) were closely monitored.

### 4.2. Autologous Bone Marrow Extraction

Animals were anaesthetized and shaved over both iliac crests. The area was disinfected with an alcohol solution and sterile drapes were applied. After skin incision, the iliac crest was punctured with a bone marrow biopsy needle for children (Jamshidi, CareFusion, Kelberg, Germany). Aspiration was done with a 20 mL syringe filled with 1 mL heparin solution (Heparin-Na 5000 IU/mL, B. Braun Medical AG, Emmenbrücke, Switzerland). Then, 6–10 mL of bone marrow were aspirated. The wound was then closed with a single stitch suture (Prolene 5/0, Ethicon, Somerville, NJ, USA) [9].

### 4.3. Bone Marrow Stromal Cell Cultures

Bone marrow aspirates were diluted 1:10 with erythrocyte lysis buffer (8.29 g/L NH_4_Cl, 1 g/L KHCO_3_, 37.2 mg/L EDTA, pH 7.3, all chemicals from Sigma-Aldrich, Fluka Chemie AG, Buchs, Switzerland) and incubated at room temperature for 5 min. After centrifugation, nucleated cells were resuspended in culture medium and counted manually in a Neubauer chamber. Plastic adhesion and proliferation of fibroblast-like cells was considered selective for MSC. Culture medium consisted of alpha-MEM, 10% fetal bovine serum, 1% penicillin-streptomycin-glutamate, 10 mM HEPES, 1 mM sodium pyruvate (all from Gibco, Invitrogen, Basel, Switzerland) and 5 ng/mL FGF-2 (R&D Systems, Wiesbaden, Germany) [30,31].

All cell cultures were performed in a humidified atmosphere at 37 °C and 5% CO_2_. For initial expansion, 10^5^ nucleated cells per cm^2^ were plated and medium was changed twice per week. When cultures were sub-confluent, cells were harvested with 3 mg/mL collagenase (Worthington Biochemical Corporation, Lakewood, NJ, USA) followed by 0.5 mg/mL trypsin (Gibco, Basel, Switzerland). Collagenase was deemed necessary because of excess extracellular matrix formation in the cultures. Cells were counted and re-plated for a second expansion step at 10^3^ cells per cm^2^.

### 4.4. Colony Forming Unit Assay

From the initial bone marrow aspirate, 3500 nucleated cells per cm^2^ were seeded on tissue culture dishes and cultured for 14 days. Cell colonies were fixed with 4% buffered formaldehyde, stained with 1% methylene blue (Fluka Chemie, Buchs, Switerland) and counted. Counts were divided by the total number of seeded cells to receive the percentage of colony forming units.

### 4.5. Preparation of Seeded Decellularized Bone

To generate the tissue engineered osteogenic grafts, cylinders of decellularized trabecular bone (Tutobone, Tutogen Medical GmbH and RTI Biologics, Neunkirchen, Germany) with a diameter and height of 7 mm were used. Tutobone is produced from bovine cancellous bone by the patented Tutoplast process. The scaffolds were placed in a custom-made silicon mold for cell seeding, which allows rolling in one axis. 2 × 10^6^ cells were suspended in 200 μL of 100 mg/mL fibrinogen (Baxter AG, Volketswil, Switzerland), applied to the scaffolds in the silicon mold and uniformly seeded by gentle rolling in an incubator at 37 °C. After 5–10 min of soaking of the cell-fibrinogen solution 100 μL of 400 units/mL thrombin with 40 µmol CaCl (Baxter AG, Volketswil, Switzerland) were added in the mold and the rolling was continued until a fibrin hydrogel had formed. Seeding efficiency was evaluated by counting the leftover cells in the seeding mold. Seeding took on average less than 30 min. Engineered grafts were kept in culture medium up to 1 h until they were implanted.

For the distribution of cells on the scaffold, an MTT staining was performed by cutting the grafts in half and incubating them for 2 h in 50 ug/mL MTT (Sigma-Aldrich) in phenol-free DMEM (Gibco). Fotographs were taken under a Carl Zeiss Stemi DV4 stereo microscope.

### 4.6. Prevascularization and Orthotopic Implantation

For the implantations, animals were randomly split into 3 groups with 3 animals each. In the pedicle 2-step group, scaffolds were first wrapped in a panniculus carnosus muscle flap, which was axial supplied by a vessel from the axillary artery. After placing the engineered graft the muscle flaps were then re-inserted subcutaneously and *in vivo* vascularization of the graft was allowed to form for 6 weeks. In a second operation the wrapped scaffold was rotated to an orthotopic segmental defect in the humerus while keeping the vessels connected to allow continuous blood flow. In the pedicle 1-step group the same procedure was performed, except that the wrapped scaffolds were immediately rotated to an orthotopic defect without leaving time for prevascularization. In the direct group, seeded scaffolds were placed into the orthotopic defect without prevascularization (see Figure 5A,B).

### 4.7. Muscle Flap Preparation

After induction of anesthesia, shaving and disinfection, the skin above the shoulder was cut. A panniculus carnosus muscle flap was prepared with ultimate care not to damage the contained vessels and nerves. The axillary vessel was identified and the scaffold was wrapped in this part of the muscle flap. The wrapped muscle was sutured with a running mattress suture (Biosyn 4/0, Covidien, Ireland). To prevent ingrowth of tissue and vessels from outside, the whole graft was wrapped in a semipermeable nylon-silicone membrane (Biobrane, Smith & Nephew, Marl, Germany), leaving the vascular pedicle free. In the pedicle 2-step group the graft was re-inserted and the wound was closed (Prolene 5/0, Ethicon).

### 4.8. Orthotopic Segmental Defect

After preparation of the animal, the skin over the sulcus bicipitalis lateralis was cut and the humerus exposed through blunt dissection while preserving the radial nerve. A midshaft segmental defect of 7 mm (5%–10% of total bone length) was created with an oscillating saw (Colibri, Synthes, Switzerland). For both pedicle groups, the graft was moved to the defect through a subcutaneous tunnel while taking care not to damage the vascular pedicle. A patent pedicle was insured through visual inspection of a red, warm and pulsating graft. In the direct group, the seeded scaffold was directly inserted. In all cases, a press-fit fixation was performed and a 2 mm locking compression plate was installed with 4 bicortical angular stable screws (2.0 LCP plate and screws, Synthes, Switzerland). The skin was sutured and no splints or bandages were applied.

### 4.9. Radiological Follow-up

Starting two weeks after implantation, radiographs were taken in two planes every month to evaluate consolidation and osteosynthesis stability. Final radiographs two weeks before sacrifice were used to quantify callus formation. Callus width immediately proximal and distal to the defect was measured and an average calculated. Care was taken to use images where the plate and screws were identically oriented.

### 4.10. Explantation and Microtomography

Twelve weeks after orthotopic implantation, the animals were anesthetized and euthanized with an overdose of 100 mg/mL pentobarbital. The whole humerus was explanted and the osteosynthesis material removed without disrupting the implant site. Samples were fixed overnight in 4% buffered formaldehyde and then stored in PBS. Microtomography was performed using a μCT 40 (Scanco Medical AG, Brüttisellen, Switzerland). Radiographs were taken at 60 kV and 160 μA every 0.25° for a rotation of 360°. Volumes were reconstructed with a voxel size of 10 μm using a modified Feldkamp back projection algorithm (Scanco Medical AG, Brüttisellen, Switzerland).

### 4.11. Histological Analysis

Samples were embedded in methylmethaacrylate (MMA) in a longitudinal orientation and cut in sections of 5–10 μm. After deplastification, slides were stained with hematoxylin and eosin (H&E), Goldner trichrome staining or tartrate resistant acid phosphatase (TRAP). Sections were analyzed and photographed under a Nikon Olympus BX61 microscope. Quantifications were performed by 2 blinded observers using the ImageJ software [32].

For necrotic core quantification, two representative non-consecutive midline sections from different depths stained with H&E were selected for each sample and scanned. The necrotic area was manually selected based on cellular and tissue morphology. Necrosis was defined as liquefied tissue and either cellular debris or cells with pyknotic nuclei. Resulting areas were normalized and represented as fold of the pedicle 2-step necrotic area for easier comparison.

For vessel quantification, one representative midline section from a central depth was chosen for each sample and 24 high-power fields covering the entire section were analyzed. Vessels were identified based on red staining of contained erythrocytes. Care was taken to avoid counting unspecific hemorrhages.

### 4.12. Statistical Analysis

Data was analyzed by one-way ANOVA with a *post-hoc* Bonferroni test and linear regression. The level of significance was set to *p* < 0.05. Graphpad Prism 5.1 was used for all analyses.
